# Low Subcritical CO_2_ Adsorption–Desorption Behavior of Intact Bituminous Coal Cores Extracted from a Shallow Coal Seam

**DOI:** 10.1021/acs.langmuir.2c02971

**Published:** 2023-01-20

**Authors:** Shakil A. Masum, Sivachidambaram Sadasivam, Min Chen, Hywel R. Thomas

**Affiliations:** Geoenvironmental Research Centre (GRC), School of Engineering, Cardiff University, The Queen’s Buildings, The Parade, Cardiff CF24 3AA, U.K.

## Abstract

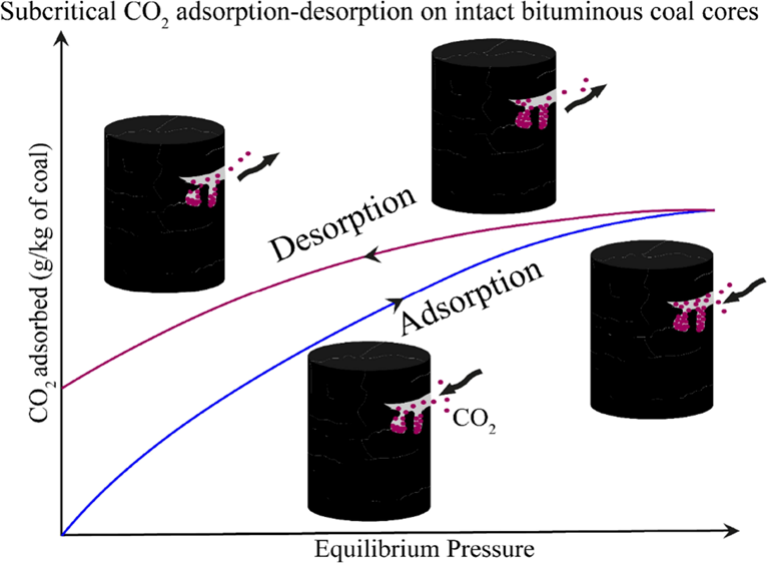

This study focuses on improving fundamental understanding of low, subcritical CO_2_ adsorption–desorption behavior of bituminous coals with the aim to evaluate the utility of shallow-depth coal seams for safe and effective CO_2_ storage. Comprehensive data and a detailed description of coal–CO_2_ interactions, e.g., adsorption, desorption, and hysteresis behavior of intact bituminous coals at CO_2_ pressures <0.5 MPa, are limited. Manometric sorption experiments were performed on coal cores (50 mm dia. and 30- or 60-mm length) obtained from a 30 m deep coal seam located at the Upper Silesian Basin in Poland. Experimental results revealed that the adsorption capacities were correlated to void volume and equilibrium time under low-pressure injection (0.5 MPa). The positive deviation, observed in the hysteresis of adsorption–desorption isotherm patterns, and the increased sample mass at the end of the tests suggested CO_2_ pore diffusion and condensation. This behavior is vital for assessing low-pressure CO_2_ injection and storage capabilities of shallow coal seams where confining pressure is much lower than that of the deeper seams. Overall, CO_2_ adsorption depicts a type II adsorption isotherm and a type H3 hysteresis pattern of the IUPAC classification. Experimental results fitted better to the Brunauer–Emmett–Teller model than the Langmuir isotherm model. CO_2_ adsorption behavior of intact cores was also evaluated by characteristic curves. It was found that Curve I favored physical forces, i.e., the presence of van der Waals/London dispersion forces to describe the coal–CO_2_ interactions. However, analysis of Curve II indicated that the changing pressure-volume behavior of CO_2_ in the adsorbed phase, under low equilibrium pressures, cannot be ignored.

## Introduction

Deep, un-mineable coal seams are usually considered as CO_2_ storage reservoirs. Although coal-seam CO_2_ storage capacity increases with the reservoir depth, gas injection operations at a deeper subsurface suffer from technical challenges and limitations which question the viability of exploiting such coal seams. For example, swelling-induced coal permeability and gas injectivity loss, driven by CO_2_ adsorption in coal under elevated confining pressures, are often reported in field tests and laboratory experiments.^1−3^ This results in under-utilization of coal resources and requires stimulation or fracturing of coal deposits for facilitating gas injection and storage. In addition, coal seams at greater depths are usually found to be intact and of high methane content. Following the announcement of the Global Methane Pledge, at the COP26 in Glasgow, exploitation of such deposits is deemed controversial, and under the European Union (EU) Green Deal further use of intact or virgin coal seams in Europe is planned to be restricted, as methane is more potent greenhouse gas than CO_2._^4^ In this context, there is an urgent need for testing the potentials of shallow coal seams for safe and effective storage of CO_2_, which is also the overarching aim of the current study. Generally, coal mines or coal-rich regions (especially in Europe) are collocated with clusters of large point-source CO_2_ emissions and far from offshore CO_2_ storage sites.^5^ The abandoned or decommissioned mines have the potential to be retrofitted as CO_2_ storage units enabling their continuous operation on reducing atmospheric CO_2_ emissions and providing economic incentives.^6^ Since the decline of coal mining, shallow seams are becoming increasingly available for new low carbon developments, and the utility of such mines for CO_2_ storage is currently investigated under the EU funded ROCCS project.^7^

Mechanisms of CO_2_ adsorption or coal–CO_2_ interactions are important to estimate the storage capacity of specific coal seams and understand its storage behaviors. CO_2_ storage potential is evident in all coal ranks, but the adsorption capacity varies with rank, moisture content, swelling characteristics, porosity, temperature, and operating pressures.^8,9^ Numerous studies have focused on both supercritical and subcritical CO_2_ injection in high- and low-rank coals, e.g., anthracite, bituminous, and lignite coals. However, in this study, the focus is limited to subcritical gas injection in bituminous coal samples collected from a shallow coal-seam located at the Upper Silesian Coal Basin in Poland.

Subcritical CO_2_ adsorption in shallow coal seams is poorly understood.^10−13^ CO_2_ adsorption capacity of coal generally increases with pressures at subcritical conditions (<7.38 MPa and <304.15 K).^9,14−22^ Micropores provide most of the surface area for gas adsorption, and it is higher in high-rank coals. However, the presence of channel-like pores and matrix swelling properties influence adsorption behavior of bituminous coals.^23−25^ Ozdemir et al.^19^ estimated the adsorption capacity of eight powdered Australian bituminous coal samples at 22 °C and pressures up to 4.0 MPa, and the reported values varied between 1.07 and 1.97 mmol/g of coal. Saghafi et al.^26^ measured the adsorption capacity of 27 samples of crushed/granular bituminous and subbituminous coal at 39 °C and pressures below 6.0 MPa. The reported maximum Langmuir volumes varied from 40 to 80 m^3^ of CO_2_ per ton of coal on a dry ash-free basis. From laboratory experiments on CO_2_ sorption in granular subbituminous coal samples, under subcritical pressure (up to 1 bar) and temperature 273–298 K conditions, Abunowara et al.^27^ reported that the gas adsorption favored low temperatures and dry coal conditions. The amount of adsorbed CO_2_ varied between 0.4 mmol/g and 0.73 mmol/g. Intact coals lose their pore network upon pulverization. Meanwhile, pore structures play a crucial role in pore diffusion and condensation of CO_2_ and therefore influence the adsorption capacity of coal specimens.^23,28,29^ This effect was observed more strongly on intact bituminous coals. Bituminous coal pores are predominately mesoporous and to a lesser extent microporous.^30^ Adsorption in mesopores contributes significantly to the total adsorption in well-developed mesopore surfaces.^31−33^ Recent molecular simulation studies on pore network models reveal that the intricate mechanisms of fluid–wall and fluid–fluid interactions, which have an effect on the gas transport mechanism in porous media, have an influence on the mechanism.^34^ The network models also account for the pore-blocking effects during desorption, which are manifested as hysteresis.^35^ Surface chemistry of carbonaceous materials can play an important role in gas adsorption. For example, functional groups, such as carboxyl and hydroxyl groups, accommodate CO_2_ molecules at the sorption sites via surface polarity or molecular bonds.^36,37^

Pone et al.^24^ measured the sorption capacity of bituminous samples, 2.5 cm diameter and 6.3 cm length, and reported adsorption of 61.6 g of CO_2_/kg of coal compared to 52.8 g of CO_2_/kg of powdered coal at 6.9 MPa equilibrium pressure. Existing literature studies largely focused on powdered or intact fragments, or small coal cores, and information on large intact samples is limited.^24,38^ Difficulty in coring brittle coal to obtain representative coal samples and a prolonged period to reach thermodynamic equilibrium in laboratory experiments are the possible reasons for data scarcity.^38^ Therefore, detailed laboratory investigations are essential to improve understanding of subcritical CO_2_ adsorption in bituminous/subbituminous coals. Intact coal samples, extracted from a target coal deposit, better preserve fractures, microfractures, and porosity information that are crucial for accurate estimation of CO_2_ storage and improved understanding of the fate of stored CO_2_ in that coal deposit.

Along with adsorption, CO_2_ desorption behaviors are also important to assess containment of stored CO_2_ at the postinjection stage.^25^ The CO_2_ adsorption–desorption isotherm is not fully reversible.^39^ The positive deviations observed in desorption isotherms are commonly attributed to CO_2_ pore trapping, swelling, and shrinking of coal matrices.^19,40^ Many experimentally derived isotherms of CO_2_ adsorption on coal have been compared to the International Union of Pure and Applied Chemistry (IUPAC) classified isotherms. The analyses of adsorption isotherms and hysteresis patterns of^19,27,41,42^ suggest that CO_2_ adsorption follows a combination of type II (BET type, which includes the Langmuir monolayer) with H1 and H3 hysteresis loops, explained by Sing et al.^43^ and Thommes et al.^44^ Isotherm patterns and hysteresis behavior of intact bituminous samples subjected to subcritical CO_2_ injection should be examined and compared with the IUPAC classification.

In this work, CO_2_ adsorption–desorption experiments were carried out on large, intact bituminous coal samples under subcritical pressure and temperature (298.15 K) conditions. Existing literature studies on subcritical CO_2_ injection tests are conducted largely in the pressure ranges around 0.5–7.0 MPa, and there is significant lack of data, below 0.5 MPa pressures, and understanding of the coal–CO_2_ interactions at lower pressures. Therefore, the experiments in this study are conducted mainly between 0.1 and 0.5 MPa CO_2_ injection pressures. However, additional experiments are conducted at intermediate pressures, i.e., from 0.5 to 4.0 MPa, on the large core (50 mm dia. and 60 mm length), and, from 0.1 to 1.2 MPa, on the powdered coal sample for comprehensive analysis and improved understanding of the observed results. The experiments in this study were conducted allowing a longer duration of equilibrium times than existing experiments in literatures. The samples were acquired from a shallow level seam in the Experimental Mine Barbara (EMB), Mikołów, Poland. The “seam-310” in EMB is located at 30 m below the surface and identified as the target seam for a pilot CO_2_ injection test under the EU-RFCS funded ROCCS project. Subcritical CO_2_ adsorption of the powdered sample was investigated, and the preferential sorption behavior of intact coal was tested by injecting a mixture of 20% CH_4_: 80% CO_2_ gas mixture. The aim of this study is to measure adsorption capacity and adsorption–desorption hysteresis of the intact bituminous coal samples which will eventually support estimation of the storage capacity of the target seam and comprehensive understanding of the state of stored CO_2_ at the postinjection stage. The experimental data were fitted to the Langmuir (monolayer adsorption) and Brunauer–Emmett–Teller or BET (multilayer adsorption) isotherm models. The thermodynamics of adsorption was also investigated. Although widely recognized practice is to fit coal-gas adsorption data to Langmuir and BET models, it should be emphasized that these isotherm models do not account chemical potential, surface interfacial forces, and the *P*–*V*–*T* behavior of adsorbed and free gas molecules. The isotherm models generally assume that the adsorbent is rigid, and its surface and pore volume remain unchanged despite coal being a nonrigid porous material whose surface and pore volumes alter upon adsorption.^45^ Therefore, to provide a better insight into coal–CO_2_ interaction mechanisms, CO_2_ adsorption on intact bituminous coal cores is evaluated using characteristic curves.

## Materials and Methods

### Manometric Sorption Apparatus Setup

**Figure 1:**
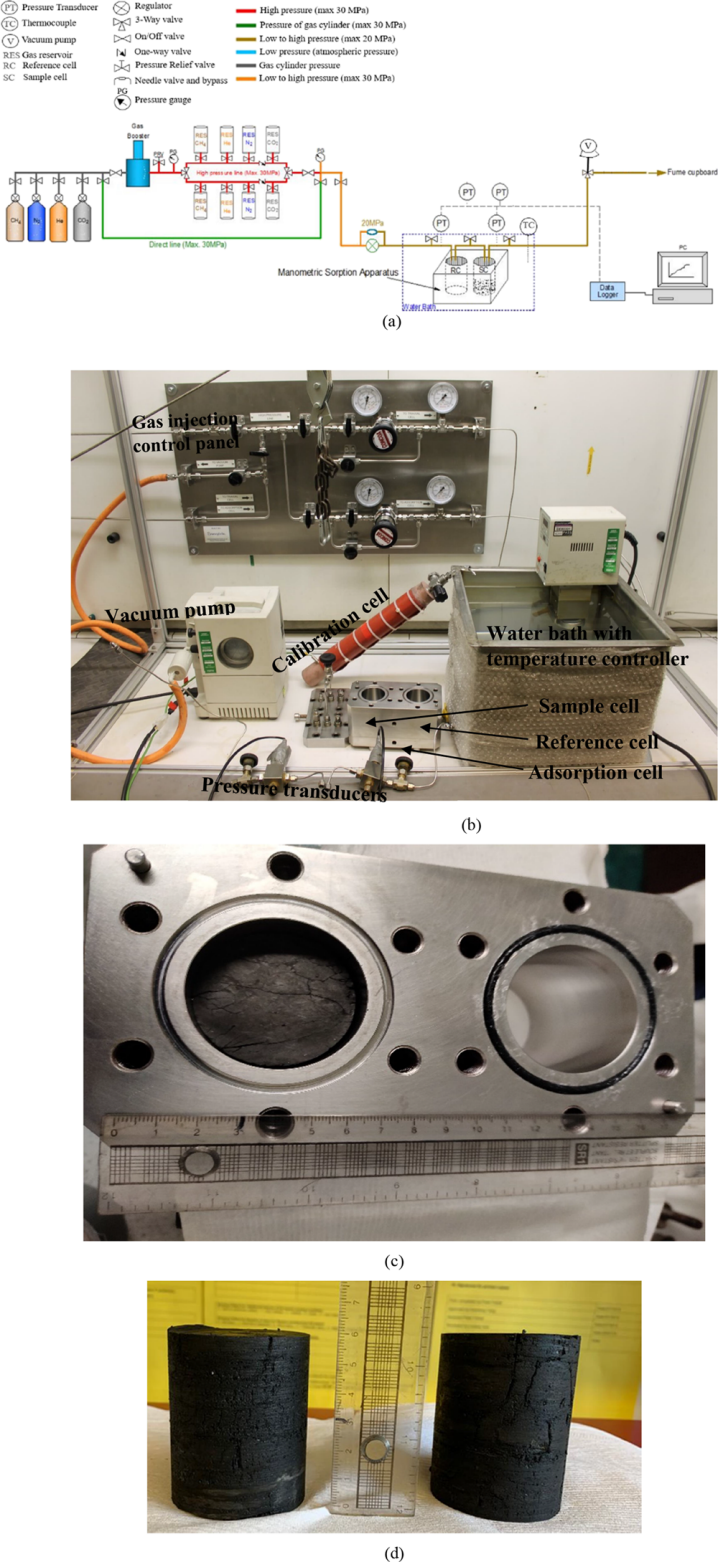
(a) Flow chart of the manometric adsorption cell experimental setup, (b) main components of the experimental setup, (c) core sample loaded in the adsorption cell, and (d) photograph of core samples drilled from the large coal blocks obtained from the Experimental Mine Barbara, Mikołów, Poland (EMB coal). Sample diameter 50 mm and length 60 mm.

The CO_2_ adsorption–desorption experiments were performed using a manometric adsorption cell (GDS Instruments UK). The apparatus is designed to sustain pressures up to 20 MPa and temperatures up to 338 K (65 °C). The entire system diagram is shown in Figure 1a–c. The flowchart of the instrumentation (Figure 1a) and its main components are described below. The apparatus mainly contains:

(i) A manometric unit consists of a reference cell (RC), where a known amount of gas is stored, and a sample cell (SC), where an adsorbent is placed.
(ii) Needle valves to connect and isolate the RC and SC.
(iii) Pressure transducers and data loggers to monitor pressure and communicate with a computer.
(iv) A temperature-controlled water bath to maintain the system temperature at 298.15 K.
(v) A calibration cell (of volume = 0.0004892 m^3^) for the helium pycnometry (He-pycnometry) test for determining the void volumes of RC and SC. The calibration cell temperature was maintained independently at 298.15 K.

To load the samples, a 50 mm dia. filter paper of pore diameter of 0.25 μm was placed at the bottom of both reference and sample cells. Following that, the sample cell was loaded with core samples (Figure 1b). O-rings were installed with a vacuum seal gel (applied), and a 55 mm dia. filter paper with a pore diameter of 0.25 μm is placed at the top of both cells to avoid particles from entering and clogging the high-pressure line. The entire system is gas sealed by the top lid of the adsorption cell. The adsorption cell was placed in a water bath maintained at a temperature of 298.15 K. A constant water level is maintained to avoid the components containing CO_2_ to be exposed to atmospheric temperature. The volume available for the gas in the sample cell with and without coal sample loaded is estimated by the He-pycnometer method explained in the Supporting Information.

### Sample Preparation

The proximate and ultimate analyses of the coal are presented in Table 1. The proximate and ultimate analyses were carried out in compliance with British Standards Institution (BSI) and American Society for Testing Materials (ASTM) specifications (BS 1016-104.3:1998, BS 1016-104.4:1998, BS 1016-104.1:1998, BS 1016-104.1:19999, BS 1016-106.1.1:1996, BS 1016-106.4.2:1996, and ASTM D3302/D3302M, Green and Perry 2019 and ASTM D388–99). The coal exhibits a low moisture content (‘as received’ 7.54 wt % and ‘analytical’ 6.39 wt %) and a high volatile content (33.94%). The carbon content is approximately 71.5%, and the maximum ash content is 15.56%. Reflectance of vitrinite is 0.57% ± 0.03%. The coal is classified as low-rank bituminous type coal. The estimated He-density of intact and powdered samples was 1389 ± 40 and 1358 kg/m^3^, respectively.

**Table 1:** Proximate and Ultimate Analysis of the EMB Coal Specimen^a^

parameter	value
as received	
moisture (%)	7.54
ash (%)	15.56
S total (%)	0.51
calorific value (%)	21,708
analytical	
moisture W^a^ (%)	6.39
ash A^a^ (%)	16.52
volatile matter V^a^ (%)	33.94
calorific value A^a^ (%)	230,192
C^a^ (%)	71.5
H^a^ (%)	3.70
N^a^ (%)	0.87
S^a^ total (%)	0.54
S^a^_c_ (%)	0.54
O^a^ (%)	14.03

a Oxygen calculated as: (O^a^) = 100 – (W^a^) – (A^a^) – (C^a^) – (H^a^) – (S_c_^a^) – (N^a^) %.

Intact core samples were drilled from the blocks using a core drill machine containing a diamond saw tip of 50 mm internal diameter (Figure 1d). Intact samples of two different lengths, 30 and 60 mm, were tested in the experiments. To obtain the powdered samples, ground pulverized coal was passed through a 63 μm diameter mesh.

### Experimental Methods of Adsorption and Desorption Tests

A known mass of coal sample (*m*_s_) is placed in the SC and degassed under vacuum to remove trapped gases from the sample. The void volume (*v*_d_) that is available for the gas in RC and SC can be approximated by the He-pycnometry method as follows:^46−48^
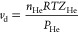
1where *n*_He_ is the number of moles of injected He (mol), *P*_He_ is the pressure of He (kPa), *Z*_He_ is the compressibility factor of He, and *R* is the universal constant = 8.314 Pa m^3^/K/mol and *T* is the absolute temperature = 298.15 K.

A known amount of CO_2_ was injected into the RC and expanded into the SC where it adsorbed on the adsorbent (coal) and progressed toward equilibrium. The difference between the amount of CO_2_ in the gas phase at equilibrium (*n*_eq_^CO_2_^) and the known amount (*n*_t_^CO_2_^) injected into the RC was measured to estimate the amount of CO_2_ adsorbed in the coal samples. The pressure in the RC was increased progressively in stages from 0.1 to 4.5 MPa. Due to the pressure being below the 6.1 to 6.4 MPa region, where liquid and vapor coexist for the temperature isotherm of 298.15 K, the amount of CO_2_ injected into the RC was not adjusted for liquid formation. The equilibrium amount was estimated as follows:
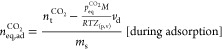

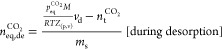
2where *n*_eq, ad/de_^CO_2_^ is the number of moles of CO_2_ at equilibrium during adsorption or desorption (mol), *v*_d_ is the void volume available to CO_2_ (m^3^), *p*_eq_^CO_2_^ is the CO_2_ pressure at equilibrium (Pa), and *Z* is the compressibility factor of CO_2_. The compressibility factor (*Z*) values were calculated using the Peng–Robinson equation of state (PR-EoS) and *m*_s_ is the mass of the coal.

CO_2_ desorption experiments were performed using similar procedures. After completing the final stage of the adsorption experiment, the pressure was decreased in RC progressively. During desorption, the pressure increased over time as the CO_2_ molecules were released from the coal surfaces. Once the equilibrium was attained, the procedures were repeated. The amount of CO_2_ released during desorption was calculated from eq 2. The change in gas pressure during adsorption and desorption was recorded every 10 s and applied in the gas law, i.e., eq 2, to calculate the desorbed amount of CO_2_. Experimental steps and calculation methods have been discussed in detail in the Supporting Information. The pressures in the adsorption cell were measured by two pressure transducers. The primary transducer in the RC was used to collect adsorption/desorption data. Before beginning the test, the secondary pressure transducer in the SC was set to similar values to the primary transducer for the purpose of validating performance and data acceptance. The pressure data deviating from the secondary transducer were plotted, and the maximum and minimum pressure deviation for any experiment was determined to be ±15 Pa (determined by multiple experimental runs from 0.1 to 6.5 MPa).

### Experimental Methods of CH_4_/CO_2_ Preferential Sorption

As mentioned earlier, the ‘seam-310’ has been de-methanized, and the concentration of CH_4_ is insignificant. However, a preferential sorption test has been conducted using a gas mixture of 80% CO_2_ and 20% CH_4_ to understand the competitive sorption behavior of the intact bituminous coal cores. The experimental methodology is similar to the adsorption–desorption test presented above. In this case, the RC was filled with the gas mixture, and the concentration of the mixture was determined using a line-connected Emerson Xtream gas analyzer. The gas mixture was then expanded to SC and allowed reaching equilibrium. Once equilibrium was reached, the RC and SC were isolated, and the gas concentration was measured to determine the amount of CH_4_ and CO_2_ adsorbed. The entire system is then evacuated through the gas analyzer to determine the concentrations of desorbing gases. In the perfect gas law equation, the partial pressure of the gases was used to determine the number of gases adsorbed/desorbed. The coal sample was then degassed continuously for 24 h using a vacuum pump. To obtain the adsorption isotherms, the procedure was repeated four times with increasing injection pressure.

### Experimental Program

Since the coal samples were procured from a shallow coal seam at 30 m below the surface and the in situ pilot test would be carried out by subcritical CO_2_ injection, the experiments were designed for pressures ranging from 0.1 to 0.5 MPa. However, for comprehensive analysis and improved understanding of the adsorption behavior, in certain experiments, the pressure was extended up to 4 MPa. The experimental program is outlined in Table 2.

**Table 2:** Experimental Program

sample	experiment	conditions^a^	tests
EMB1: 50 mm dia. and 60 mm length	EXP1-A	0.5 to 4.0 MPa injection pressure 298 K	He adsorption
			CO_2_ adsorption
	EXP1-D	3.6 to 0.085 MPa equilibrium pressure 298 K	CO_2_ desorption
	EXP2-A	0.1 to 0.5 MPa injection pressure 298 K	He adsorption
			CO_2_ adsorption
	EXP2-D	0.41 to 0.041 MPa equilibrium pressure 298 K	CO_2_ desorption
	EXP3	0.1 to 0.5 MPa injection pressure 298 K	He adsorption
			CO_2_/CH_4_ adsorption
EMB2: 50 mm dia. and 30 mm length	EXP4-A	0.1 to 0.5 MPa injection pressure 298 K	He adsorption
			CO_2_ adsorption
	EXP4-D	0.33 to 0.08 MPa equilibrium pressures 298 K	CO_2_ desorption
EMB3: powdered coal	EXP5	0.1 to 1.2 MPa injection pressure 298 K	He adsorption
			CO_2_ adsorption

a Adsorption test pressures represent injection pressures while the desorption test represents equilibrium pressures.

## Adsorption Theory

### Evaluation of CO_2_ Adsorption by Langmuir and BET Models

CO_2_ adsorption on the bituminous coal samples was evaluated by Langmuir and BET isotherm models. The Langmuir model describes both physical and chemical adsorption based on a monolayer adsorption on a homogeneous surface. However, the BET model describes monolayer and multilayer adsorption, and it is based on the assumption that the heat of adsorption is equal to the heat of condensation and it is the same on different layers.^49^ Since, at subcritical temperatures, the type II isotherm overlaps with type I indicating the formation of multilayers, the BET model has been adopted here. The nonlinear form of the Langmuir model^47^ is as follows:
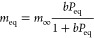
3where *m*_eq_ is the mass of adsorbed gas at a given equilibrium pressure (g/kg), *P*_eq_ is the equilibrium pressure (Pa), *m*_∞_ is the maximum adsorption capacity (g/kg), and *b* is the Langmuir parameter, which is also reciprocal of half-loading pressure (Pa^–1^). The *m*_∞_ and *b* values, obtained from the nonlinear regression analysis, were used in eq 3 for validating the experimental data.

The thermodynamic parameters, e.g., the energy of adsorption (Δ*H*_ad_) and Gibbs free energy (Δ*G*_ad_^0^) were obtained from model fitting exercises. The number of molecules hitting an active adsorption site of area σ_A_ per second is expressed as:^49^
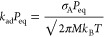
4eq 4 can be rearranged as follows:
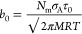
5
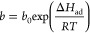
6where *k*_ad_ is the adsorption equilibrium constant, *P*_eq_ is equilibrium pressure, σ_A_ is the cross-sectional area covered by one CO_2_ molecule (2.609 × 10^–19^ m^2^), *k*_B_ is the Boltzmann constant, τ_0_ is the vibration period related to the residence time of adsorbed CO_2_ molecule (typically in the order of 10^–13^ s), *N*_m_ is the number of molecules adsorbed (related to *m*_∞_), *M* is the molar mass of CO_2_ (44.01 g/mol), and *b*_0_ is the pre-exponential of the Langmuir constant *b*.

Gibbs free energy (Δ*G*_ad_^0^, kJ/mol) is calculated as:
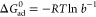
7

The BET model assumes multilayer adsorption, and it can be expressed as:^50^
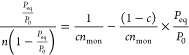
or
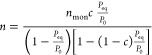
8where *n* is the adsorbed moles (mol/kg), *n*_mon_ is the number of moles to cover monolayer adsorption (mol/kg), *P*_0_ is the saturation pressure of CO_2_ at critical condition (Pa), and *c* is a dimensionless parameter related to the energy of adsorption and condensation, , where *Q*_1_ is the equilibrium constant of adsorption on the bare surface (monolayer adsorption) (J/mol) and *Q*_2_ is the equilibrium constant for physisorption of the overlaying layers (treated as bulk fluid) (J/mol). The *c* and *n*_mon_ values were acquired from the nonlinear regression analysis and were used in eq 8 for validating the experimental data.

The present study also calculated the amount of adsorbed CO_2_ over a specific surface area following the work of Yang^51^ and Tien.^52^ The surface area of one mole of CO_2_, occupied in the liquid state, is calculated as:
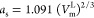
9where *a*_s_ is the effective surface area covered by 1 mol of CO_2_ (m^2^/mol), and *V*_m_^L^ is the liquid molar volume of CO_2_ (m^3^/mol). The *V*_m_^L^ is calculated from the PR-EoS for the temperature pressure values of 298.15 K and 6.439 MPa, respectively (*V*_m_^L^ = 70.7 cm^3^/mol). The number 1.091 is the packing factor of 12 neighboring molecules in a bulk liquid and six on a plane.^51^ The specific surface area is then calculated as:
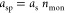
10where *a*_sp_ is the specific surface area (m^2^/kg).

### Evaluation of CO_2_ Adsorption by Characteristic Curves

CO_2_ adsorption on the bituminous coal samples was evaluated using characteristic curves. The curves represent physical attraction between a large body of adsorbent and a small gas molecule (Characteristic curve I),^49,51−55^ and variation of adsorbed phase molar volume due to variable equilibrium pressures (Characteristic curve II).^56^

#### Characteristic Curve I

Physical attractive forces such as the van der Waals and the London dispersion forces play important roles in coal–CO_2_ interactions. The potential theory postulates that the thickness and number of moles spread over a specific surface area of an adsorbent are affected by the state of the chemical equilibrium and the physical attractive forces between the sorbent and the sorbates.^49,53^ Therefore, the chemical potential of the adsorbed phase and the free gas phase equals
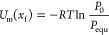
11

For a gas molecule, attracted to a surface by van Der Waals force, eq 11 can be rewritten as:^47,50^
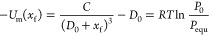
12

Then, the number of molecules adsorbed per unite area can be defined as:
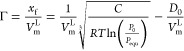
13where *U*_m_ is the molar internal energy (J/mol), Γ is the moles adsorbed over a specific surface area (mol/m^2^), *x*_f_ is the thickness of the liquid-like adsorbed layer related to density and effective surface area of adsorbents (m), *C* is a constant specific to the solid–gas adsorption system and related to the internal energy of the surface (involving physical attraction forces) (Jm^3^/mol), and *D*_0_ is the effective radius of the CO_2_ molecule (3.3 × 10^–10^ m). Eq 11 is used to predict the number of CO_2_ moles adsorbed over a unit surface area using the constant *C* that relates the physical attraction forces of coal surfaces exerted on CO_2_ molecules.

#### Characteristic Curve II

The characteristic curve assumes that the molar volume of adsorbed phase (MVAP) CO_2_ varies with adsorptive gas pressure. The MVAP reaches liquid-like density inside the pores and surfaces of coal at an intermediate pressure (≤0.5 MPa). This type of adsorption is limited to the fugacity of the adsorbed phase and the fugacity of the gas phase, especially, when they are equal.

To evaluate the hypotheses, the MVAP of CO_2_ was calculated and correlated with the molar volume of a monolayer predicted by the BET model. The variation of molar volume ratio was plotted against the fugacity ratio of the gas phase and adsorbed phase. The *v_m_^ad^* was calculated as:
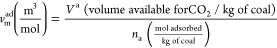
14where *V*^a^ is the volume available for CO_2_ in per kg of coal, which is equal to the void volume of the sample + maximum volume occupied on the external surface area (assuming no multilayer on the surface) + connected and unconnected pore volume (1.5% of the bulk volume of the sample), and *n*_a_ is the amount of CO_2_ adsorbed/kg of coal (mol/kg).

Then, the adsorbed phase molar volume was plotted against the fugacity ratio of the adsorbed phase and gas phase:
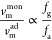
or
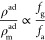
15

where *v*_m_^mon^ is the molar volume of monolayer coverage (m^3^/mol), ρ^ad^ and ρ_m_^ad^ are the densities of the adsorbed phase and molar volume of monolayer coverage (mol/m^3^), respectively, and *f*_g_ and *f*_a_ are the fugacity of the gas phase and adsorbed phase (MPa), respectively.

## Results and Discussion

### Adsorption–Desorption Isotherm and Hysteresis Behavior

**Figure 2:**
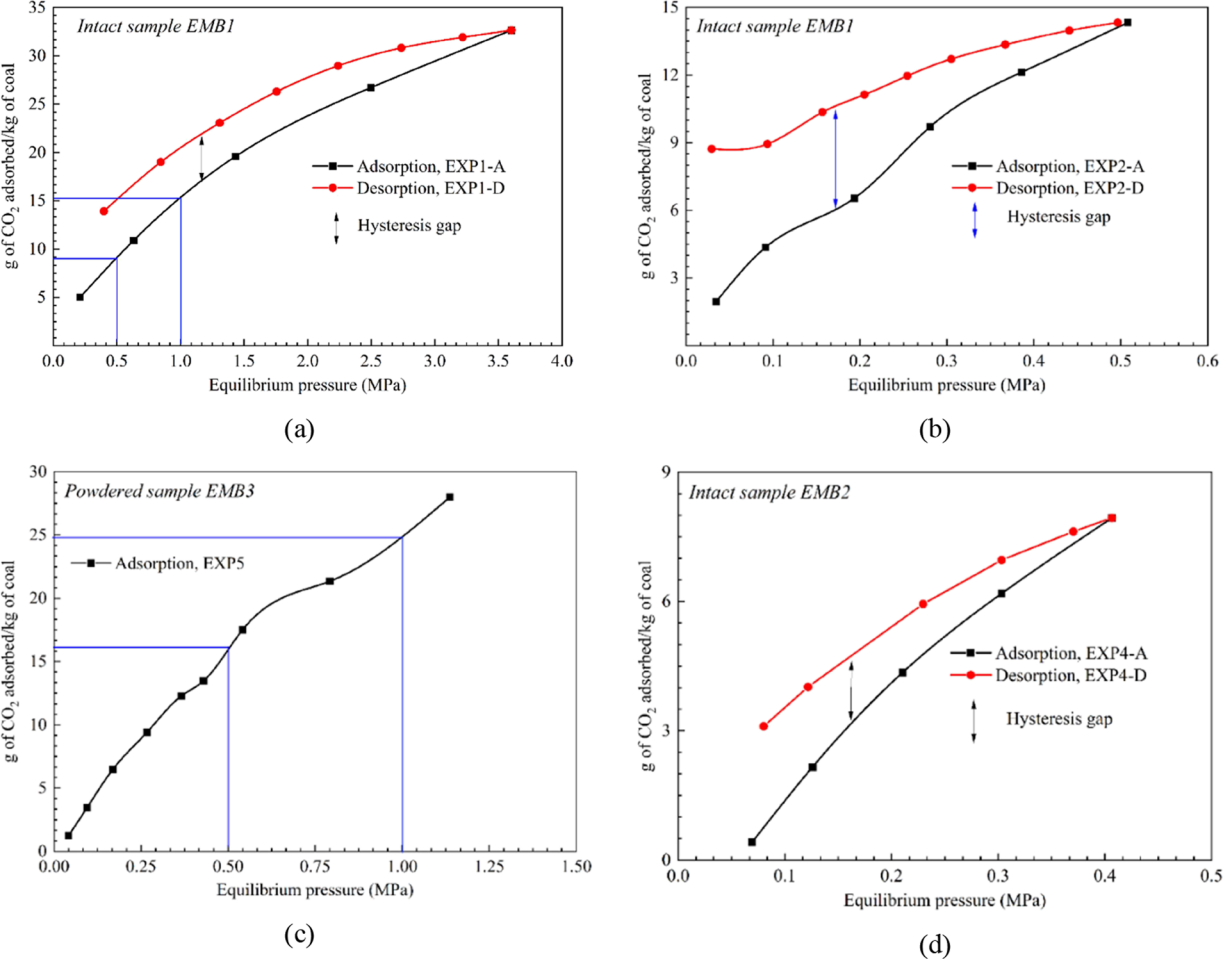
CO_2_ adsorption–desorption isotherms of intact sample EMB1 (50 mm dia. and 60 mm length) for injection pressure (a) up to 4.0 MPa, and (b) up to 0.5 MPa. (c) CO_2_ adsorption isotherm of powdered sample EMB3 for injection pressure up to 1.2 MPa. (d) CO_2_ adsorption–desorption isotherm pattern of the intact sample EMB2 (50 mm dia. and 30 mm length) for the injection pressure up to 0.5 MPa.

Figure 2a,b shows the CO_2_ adsorption amount of the intact sample EMB1 under the experimental condition, EXP1 and EXP2, respectively. The observed maximum CO_2_ adsorption in EXP1 (injection pressure up to 4.0 MPa) is 32.6 g of CO_2_/kg of coal at the equilibrium pressure of 3.6 MPa, whereas in EXP2 (injection pressure up to 0.5 MPa) it is 14 g of CO_2_/kg of coal at the 0.5 MPa equilibrium pressure. Figure 2c shows the CO_2_ adsorption amount of the powdered coal sample (EMB3) for EPX5 conditions (injection pressure up to 1.2 MPa). The maximum adsorption observed in this case is 27.9 g of CO_2_/kg of coal at an equilibrium pressure of 1.13 MPa. The injection pressure was controlled for each injection step, but the stable pressure value after the adsorption attained equilibrium, noted as equilibrium pressure, was not controlled. A summary of the adsorption experiment results and observations is presented in Table 3.

**Table 3:** Summary of the CO_2_ Adsorption Amount, Equilibrium Time, and Void Volume of Core Samples

sample	experiment no	observed peak adsorption (g of CO_2_/kg of coal)	cumulative equilibrium time (h)	void volume (m^3^)	sample mass before and after experiments
EMB1	EXP1-A	32.6	259^a^	4.5 × 10^–6^	before: 151.61 g
		injection pressure: 0.5 to 4.0 MPa			after: 152.63 g
					increase: 0.67%
	EXP2-A	14	195	9.2 × 10^–6^	before: 140.43 g
		injection pressure: 0.1 to 0.5 MPa			after: 141.26 g
					increase: 0.55%
	EXP3	12	103	4.3 × 10^–6^	before: 145.72
		injection pressure: 0.1 to 0.5 MPa			after: 146.28
					increase: 0.38%
EMB2	EXP4-A	8	74	5.6 × 10^–6^	before: 72.61
		injection pressure: 0.1 to 0.5 MPa			after: 72.93
					increase: 0.44%
EMB3	EXP5	27.9	154^b^	3.7 × 10^–5^	
		injection pressure: 0.1 to 1.2 MPa			

a It took 80 h to reach equilibrium for 0.5 MPa injection then 40 h to 1 MPa injection pressure.

b Up to 154 h to reach equilibrium at 0.5 MPa injection pressure.

The adsorbed concentrations at the 0.5 MPa and 1.0 MPa equilibrium pressures in powdered coal sample are 16.5 and 25.4 g of CO_2_/kg of coal, respectively. The corresponding concentrations for the intact sample (EMB1) in EXP1 are 9.2 and 15.3 g of CO_2_/kg of coal. As anticipated, the larger surface area of the powdered sample results in higher adsorption of CO_2_ than the intact coal samples. However, in EXP2 where the EMB1 sample was subjected to low injection pressure, adsorbed concentration was 14 g of CO_2_/kg of coal at 0.5 equilibrium pressure which is significantly larger than 9.2 g of CO_2_/kg of coal observed at EXP1. Comparatively, the measured void volume (Table 3) of the sample in EXP1 (4.5 × 10^–6^ m^3^) was less than that of the sample in EXP2 (9.2 × 10^–6^ m^3^). In EXP1, the initial injection pressures of 0.5 and 1 MPa took a cumulative time of 120 h to attain equilibrium pressures of 0.21 and 0.63 MPa, whereas in EXP2, it took 195 h in total to reach an equilibrium pressure of 0.5 MPa. The larger void volume and longer equilibrium time allowed more gas to be adsorbed in the coal of the same dimension obtained from the same block of coal. The powdered sample (EMB3) void volume was 3.72 × 10^–05^ m^3^ and the packing density was 1358 kg/m^3^.

The CO_2_ adsorption amount of the intact bituminous sample EMB2 (50 mm dia. and 30 mm height) is presented in Figure 2d. The sample is smaller in dimension than the EMB1 and shows reduced adsorption than EMB1. At an equilibrium pressure of 0.4 MPa, the adsorbed amount was 7.9 g of CO_2_/kg of coal which was approximately two third of the adsorbed amount of the large EMB1 sample at the same equilibrium pressure. The samples were drilled from the same block of coal. Experimental results show the influence of microfracture and pore network structure of bituminous coal on its adsorption, which exists in higher quantities in larger samples.

The intact coal samples, tested in this study, showed adsorbed CO_2_ values close to those of the powdered sample. For example, at 0.4 MPa equilibrium pressure adsorbed concentrations of EMB1(intact), EMB2 (intact), and EMB3 (powder) were 8.0 (Figure 2a)–12.5 (Figure 2b), 7.8 (Figure 2d), and 12.7 (Figure 2c) g CO_2_/kg of coal, respectively. Owing to the larger surface area of the powdered samples, they are expected to show a higher adsorption amount. However, experimental results and previous molecular simulation studies concluded that the porous nature of the bituminous coal has an important role in its adsorption capacity.^57^ An increased or similar adsorption to powder samples is expected in intact bituminous samples, as the intact samples have channel-like micropores inducing pore condensation and diffusion where the CO_2_ can be adsorbed as a whole phase.^23,24,29^ Previous experiments, conducted at low equilibrium pressures, showed a closely matching adsorption isotherm pattern for powdered and intact samples.^58^ The microfracture network in intact bituminous coal is lost upon pulverization (to prepare powder samples) which reduces the effect of pore condensation and diffusion in powdered samples and eventually its relative adsorption capacity. Similarly, in smaller intact samples, the network volume is lower which results in lower adsorption than the larger samples.

The desorption isotherm patterns of EXP1, 2, and 4 are presented in Figure 2a,b,d 6, respectively. The results indicate the CO_2_ pore trapping capabilities of the intact coal samples. As the gas desorption progresses, the hysteresis gap is widened due to the delay in the release of the trapped/adsorbed CO_2_ from the coal matrix and/ cleat system. The observed hysteresis gap for the larger sample is wider than that of the smaller sample implying that under the similar thermodynamic equilibrium conditions, larger amount of CO_2_ is trapped in the large sample. The kinetic data, published in Sadasivam et al.,^25^ also revealed that CO_2_ release occurred even after the system was evacuated to zero pressure during the desorption step-down stages. For example, the hysteresis gap at 0.2 MPa was about 4 g of CO_2_/kg of coal (EXP1, Figure 2b) for the large EMB1 sample and 1.5 g of CO_2_/kg of coal (EXP 2, Figure 2d) for the small EMB2 sample. The EMB1 sample showed a residual 8.7 g of CO_2_/kg of coal (Figure 2b) that was still adsorbed on the coal at the end of the desorption experiment, while the EMB2 sample showed a residual amount of 3.1 g of CO_2_/kg of coal (Figure 2d) for the experiments conducted in a similar pressure range, i.e., EXP2 and EXP4. This is further evident in the differences of sample mass before and after the experiments presented in Table 3. The samples mass, under these two experimental conditions, increased by 0.55 and 0.44%, respectively. However, when the same sample is subjected to a higher thermodynamic equilibrium condition, the residual amount of CO_2_ that is adsorbed and entrapped in the sample increases. For example, the differences in mass % in EXP1 and EXP2 experiments are 0.67 and 0.55%, respectively. This suggests that the residual CO_2_ entrapment increases with injection pressures. The results demonstrate the CO_2_ pore entrapment capability of bituminous coals even at low, subcritical injection pressure conditions supporting the utility of shallow coal seams as candidate CO_2_ storage reservoirs. The hysteresis observed in adsorption and desorption isotherms suggest that certain amount of CO_2_ will remain adsorbed and/entrapped within the coal despite the pressure is reduced. Additionally, by selecting a coal deposit that is surrounded by tight, impermeable host-rock (e.g., the seam “310” at the Barbara Mine, Poland) will further reduce safety concerns associated with shallow-depth coal seams. The in situ CO_2_ injection test,^7^ designed for testing the gas storage in a shallow-depth coal seams, will provide vital information on safety and effectiveness of such coal deposits with greater details.

In general, gas adsorption is physical and reversible. During the desorption experiments, the amount of gas adsorbed traces back along the isotherm patterns observed during the adsorption process. Hysteresis arises when the amount of CO_2_ adsorbed during the desorption does not match with the amount adsorbed during the adsorption at given thermodynamic equilibrium conditions. The positive deviations observed in the desorption isotherms are attributed to pore diffusion/condensation and perhaps induced by shrinkage/swelling of the bituminous coal matrix.^18,19,41,59,60^ Evidently, the reversibility of the CO_2_ adsorbed on bituminous EMB coal samples was clearly influenced by the pore condensation and diffusion of CO_2_. The nature of the observed isotherm also reveals the hysteresis pattern normally observed for narrow pore entrance (ink bottle neck), where the evaporation of the condensed gases is influenced by the shape of the pores.^43,49^

**Figure 3:**
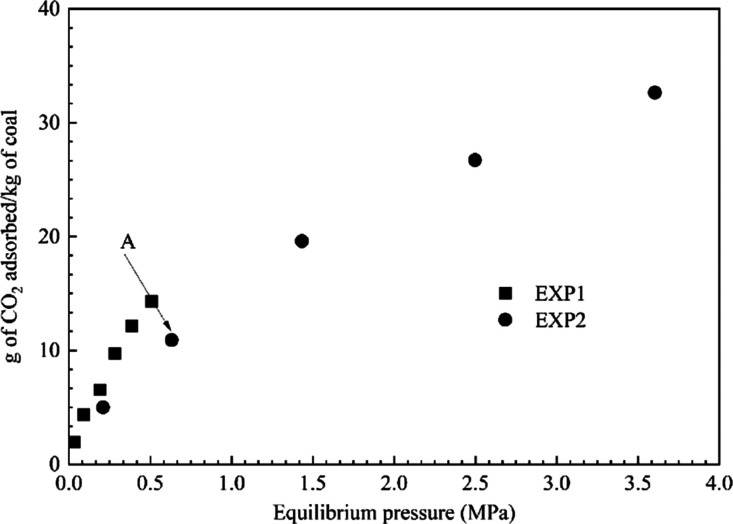
CO_2_ adsorption isotherm of intact sample EMB1 for combined EXP1 and EXP2 experiments. Equilibrium pressure ranges from 0.03 to 3.5 MPa. Sample dimension: 50 mm dia. and 60 mm length.

Figure 3 combines the adsorbed CO_2_ amount in EMB1 coal sample for all experimental pressure ranges (EXP1 + EXP2). The isotherm pattern depicts the type I or Langmuir-type monolayer pattern, described in IUPAC classification of adsorption isotherms,^43^ at low pressures. At intermediate pressures, the multilayer or type II adsorption isotherm is evident. The isotherm pattern shows a clear inflection point (marked A in Figure 3) at equilibrium pressure around 0.5 MPa and increases linearly afterward indicating multilayer adsorption. Pore condensation/diffusion can be the reason for this type of isotherm pattern. The inflection point indicates that the liquid-like molar volume begins to form way before the monolayer coverage is completed.^51^ The hysteresis patterns presented in the Figure 2a–d represent the H3 adsorption–desorption hysteresis described in the IUPAC classification.^41,42^ Similar observations were reported in previous studies that examined bituminous coal specimens. For example, Wang et al.,^61^ White et al.,^8^ and De Silva et al.^62^ explained the CO_2_ adsorption by a multilayer BET model. Harpalani et al.^41^ further explained that the linear pattern observed in the isotherm and attributed it to the pore condensation and diffusion of CO_2_ in the coal matrix and/cleat network. Observed isotherms in the present study indicated initial microporous filling followed by mesoporous occupancy that was also reported by Sadasivam et al.^25^ Adsorption in mesopores could be substantial as the fluid–wall attraction becomes prevalent, and it is reflected by steep slopes in the isotherms. Meanwhile, adsorption in mesopores might depend not only on fluid–wall interaction but also on fluid–fluid interaction, which results in capillary condensation.^32,33^ This is aligned with the type II adsorption stated by the IUPAC.

### Preferential Sorption Behavior of Coal

**Figure 4:**
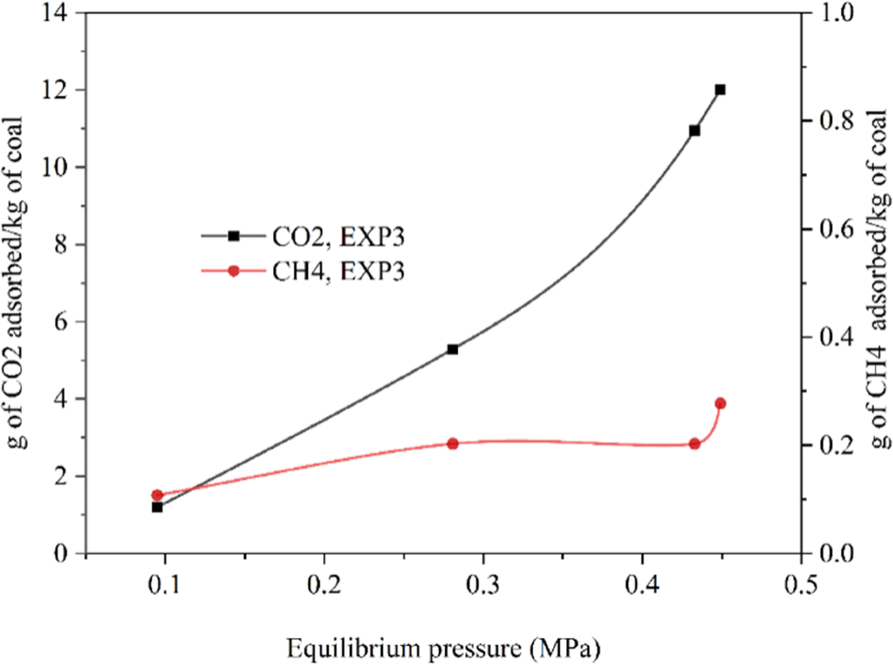
Preferential sorption behavior of intact coal for a gas mixture of 20% CH_4_: 80% CO_2._ Intact sample EMB1 (50 mm dia. and 60 mm length). Injection pressure range 0.1 to 0.5 MPa.

Figure 4 shows an adsorption of 12 g of CO_2_/kg of coal approximately at an equilibrium pressure of 0.45 MPa (EXP3), which is comparable to the amount of CO_2_ adsorbed on the sample (50 mm dia. and 60 mm length) during the pure CO_2_ adsorption i.e., EXP2. A small amount of CH_4_ adsorption was also observed during the experiment. The preferential sorption experiment was carried out in such a way that the adsorbed gases were completely desorbed to at each equilibrium pressure stage, evacuated by a vacuum pump, and restarted with a higher equilibrium pressure step. Because these procedures were time-consuming, only a limited number of data points were collected. The procedure was used to determine the volume percentage of desorbing gases in comparison to the volume percentage of injected composition. The change in the volume percentage of the gas mixture is shown in Table 4.

**Table 4:** Change in Volume Percent Observed during Preferential Sorption Experiments

equilibrium pressure, MPa	vol % CO_2_ initial	vol % CO_2_ equilibrium	vol % CH_4_ initial	vol % CH_4_ equilibrium	displaced CO_2_, vol %	displaced CH_4_, vol %
0.095	81.13	80.01	19.9	20.4		
0.28	80	71.5	20	28.55		
0.43	80.46	65.2	20.31	34.7	84.58	15.19
0.45	80.38	73.13	20.17	26.6	84.7	15.2

From previous preferential sorption experiments, Pone et al.^24^ observed the maximum sorption capacity of a bituminous coal sample around 1.6 g/kg of coal for CH_4_ and 66.01 g/kg of coal for CO_2_ at 6.9 MPa. Ottiger et al.^63^ reported a decreasing trend for both CO_2_ and CH_4_ with decreasing concentration of a specific compound in the gas mixture, and the decrease in the adsorbed amount was much stronger for methane than for CO_2_. Table 4 shows the CO_2_/CH_4_ gas mixture concentrations monitored during the preferential sorption experiment in this study. The initial gas mixture was 20% CH_4_:80% CO_2_. At equilibrium, the volume percentage of CO_2_ in the gas phase decreased and the volume percentage of CH_4_ increased compared to the initially injected gas mixture, indicating the preferential affinity of the EMB coal sample toward CO_2_. The volume percentages measured in the desorbing gas showed an inverse pattern. A gas mixture containing a higher percentage of CO_2_ was released from the adsorbed phase, confirming the isotherm pattern observed in Figure 4 (Table 4). The current study provided the quantitative and qualitative evidence of bituminous coal’s affinity to CO_2_ over CH_4_ in a much lower pressure range (0.5 MPa) to ascertain the possibilities of CO_2_ storage in shallow coal seams. From their molecular simulation studies, Brochard et al.^64^ found that coal swelling was insensitive and proportional to the CO_2_ molar fraction. The sorption behavior of the CH_4_:CO_2_ gas mixture seems similar to that of the sole CO_2_ sorption. This behavior was also observed by Lee et al.^2^ in their experiments on competitive adsorption of CO_2_ and CH_4_ gas mixtures.

### Evaluation of CO_2_ Adsorption on Coal Specimens Using Langmuir and BET Isotherm Models

**Figure 5:**
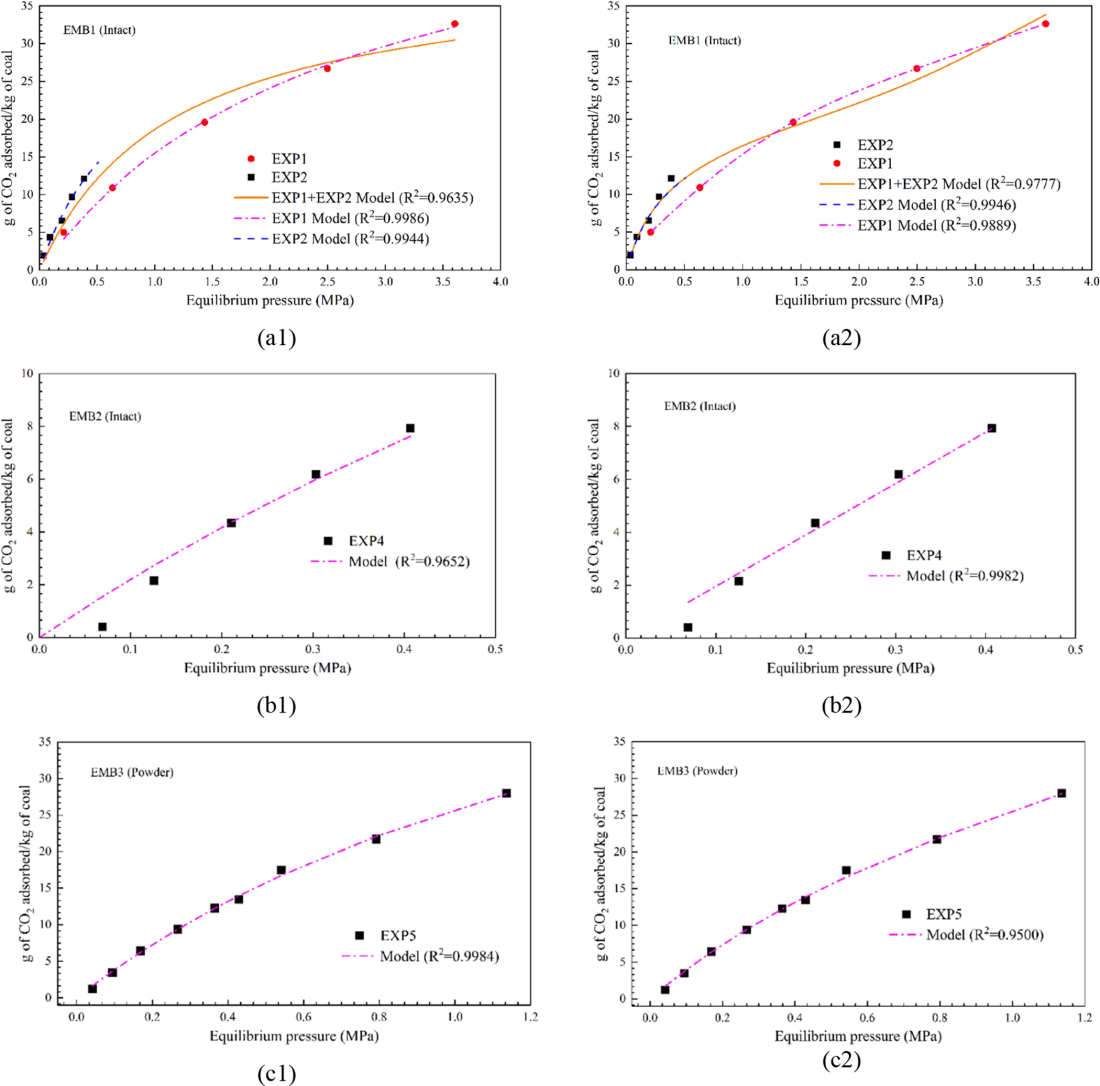
Langmuir model fit to intact (a1) EMB1, (b1) EMB2, and powdered (c1) EMB3 coal–CO_2_ adsorption data. BET model fit to intact (a2) EMB1, (b2) EMB2, and powder (c2) EMB3 coal sample data.

Figure 5a shows the fitting results of the experimental data to the Langmuir model and the BET for the intact (EMB1 and 2) and powdered coal (EMB3) samples. Along with individual EXP1 and EXP2 data, a combined model fit for EMB1 is presented in Figure 5a1,a2. The summary of the Langmuir, BET, and thermodynamic parameters obtained from the fitting exercises is listed in Table 5.

**Table 5:** Fitting Results of Langmuir and BET Models to the CO_2_ Adsorption Data

	Langmuir model fit				BET model fit			
sample	parameter, b (Pa^–1^)	maximum adsorption capacity, *m_∞_* (g of CO_2_/kg of coal)	standard Gibbs free energy, Δ*G*_ad_^0^ (kJ/mol)	energy of adsorption Δ*H*_ad_ (kJ/mol)	parameter, *c*	adsorbed monolayer coverage, *n*_mon_ (g of CO_2_/kg of coal)	specific surface area, *a*_sp_ (m^2^/kg)	*Q*_1_*–Q*_2_^a^ (kJ/mol)
EMB1 EXP1-A	3.86 × 10^–07^	55.4	–36.6	–15.56	14.43	18.83		6.60
EMB1 EXP2-A	1.31 × 10^–06^	35.57	–33.6	–19.58	13.05	27.18		6.37
EMB1 EXP1-A+ EXP2-A	8.54 × 10^–07^	40.4	–34.6	–18.21	22.67	18.16	64,826 (for *c*> 20)	7.74
EMB2 EXP4-A	6.20 × 10^–07^	37.9	–35.4	–17.57	11.0	18		5.94
EMB3 EXP5	5.74 × 10^–07^	70.7	–35.6	–15.86	7.78	40.34		5.08

a The difference between the energy of adsorption of the first layer and the subsequent liquid layers.

The Langmuir model fitting results, Figure 5a1,b1,c1, show good fits to the experiments conducted on the intact samples at low pressures (≤0.5 MPa), as well as the powdered sample, conducted in an intermediate pressure range. However, at higher pressures, the model predicted values are slightly deviated from the observed data (Figure 5a1) for the EMB1 sample. The reason for this behavior is perhaps due to the pore condensation/pore diffusion phenomena which is described in the previous section. The maximum adsorption capacity of powdered bituminous coal is 97 g of CO_2_/kg of coal and the half-loading pressure (Langmuir parameter *b*) is 3.80 × 10^–07^ Pa^–1^. The maximum adsorption capacities of intact bituminous coal samples are approximately 31 g of CO_2_/kg of coal (up to 0.5 MPa injection pressure) and 39 g of CO_2_/kg of coal (up to 4.0 MPa injection pressure). The energy of adsorption (Δ*H*_ad_) is calculated based on the Langmuir parameters obtained from the isotherm model fit. Δ*H*_ad_ values in these experiments lie between −15 and −20 kJ/mol attributing to physical adsorption. The enthalpy change of physical adsorption is in the range of −20 kJ/mol.^54^ Adsorption is spontaneous and increases with the injection pressure and reverses with reduction of pressure. However, the adsorption that took place in the narrow fractures or coal cleats was released with a time lag, which was observed during the desorption experiments. One of the reasons for the physical adsorption is that the coal surface influences the polarizability of CO_2_ molecules and induces the dipole–dipole or quadrupole type interaction with CO_2_ molecules (London dispersion forces). Therefore, it is appropriate to suggest that the surface energy holds the CO_2_ molecules on the coal surface, which is related to the internal energy of the adsorbed phase. At equilibrium, the amount of gas adsorbed is equal to the amount of desorbed gas, and the Gibbs free energy (Δ*G*_ad_^0^) is related to the equilibrium constant or the Langmuir constant (*b*). Both Δ*H*_ad_ and Δ*G*_ad_^0^ are molar quantities and increase upon the number of adsorbed moles. The Gibbs free energy for the maximum adsorption is related to the half-loading pressure *b* (Table 5). The Δ*H*_ad_ values for carbonous materials are in the range of 25–355 kJ/mol for low to high surface coverages.^65^ More specifically, the values for an 86% carbon content coal range from 25.3 to 27.3 kJ/mol,^19^ which are comparable to the estimated values of the current study.

Figure 5a2,b2, c2 shows the results of the experimental data fit to the BET model for the intact (EMB1 and 2) and powdered coal (EMB3) samples. The results show good fits to the experiments conducted on both the intact samples and the powdered sample. In comparison to the Langmuir model fit (Figure 5a1), the BET model shows better fit at higher pressures (Figure 5a2) which further confirms the multilayer adsorption with pore condensation or pore diffusion.

The dimensionless BET parameter *c* is related to the energy of multilayer adsorption and is defined as^50^
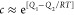
16

From this equation, the energy of condensation in multilayer adsorption can be analyzed qualitatively.

The energy of condensation is usually similar to *Q*_2_. The (*Q*_1_ – *Q*_2_) values are obtained from the *c* values (Table 5). As mentioned earlier, the *Q*_1_ values are associated with the energy of monolayer adsorption, i.e., Δ*H*_ad_. Given that at EXP1 the Δ*H*_ad_ = 21.7 kJ/mol (Table 5), the corresponding value of *Q*_2_ is approximately 15.1 kJ/mol. Please note that this is a qualitative estimation. The energy of condensation of CO_2_, according to NIST Chemistry WebBook, is about 16.7 kJ/mol^66^ (NIST Chemistry WebBook, SRD 69, 2021).

The inflection point occurred well below 1 MPa for the EMB1 specimen (Figure 3) which could be the reason for the intermediate pressure experimental results fitted well with the BET model. Since the *c*-value for the intact EMB1 sample at EXP1 + EXP2 experimental condition is more than 20, the corresponding monolayer coverage value (*n*_mon_) can be used to calculate the specific surface area (*a*_sp_) available for the CO_2_ molecules^67^ following eqs 8 and 9. For the 50 mm dia. and 60 mm long sample, the calculated specific surface area is 64,826 m^2^/kg of coal. CO_2_ adsorption is a different phenomenon than the liquid N_2_ adsorption. The CO_2_ molecules enter the unconnected pores as the coal slightly deforms during CO_2_ adsorption. The observed specific surface area by N_2_ adsorption was lower than the specific surface area measured using CO_2_ adsorption.^28^

### Evaluation of CO_2_ Adsorption by Characteristic Curves

As mentioned earlier that the Langmuir and BET models do not consider the influence of the chemical potential, and the *P*–*V*–*T* behavior of the free and adsorbed gas, CO_2_ adsorption isotherms (combined data of experiment EXP1 and EXP2 for sample EMB1) are analyzed here using characteristic curves. The curves are developed based on the potential theory of adsorption (curve I) and the adsorbed phase molar volume amounts (curve II).

#### Characteristic Curve I

**Figure 6:**
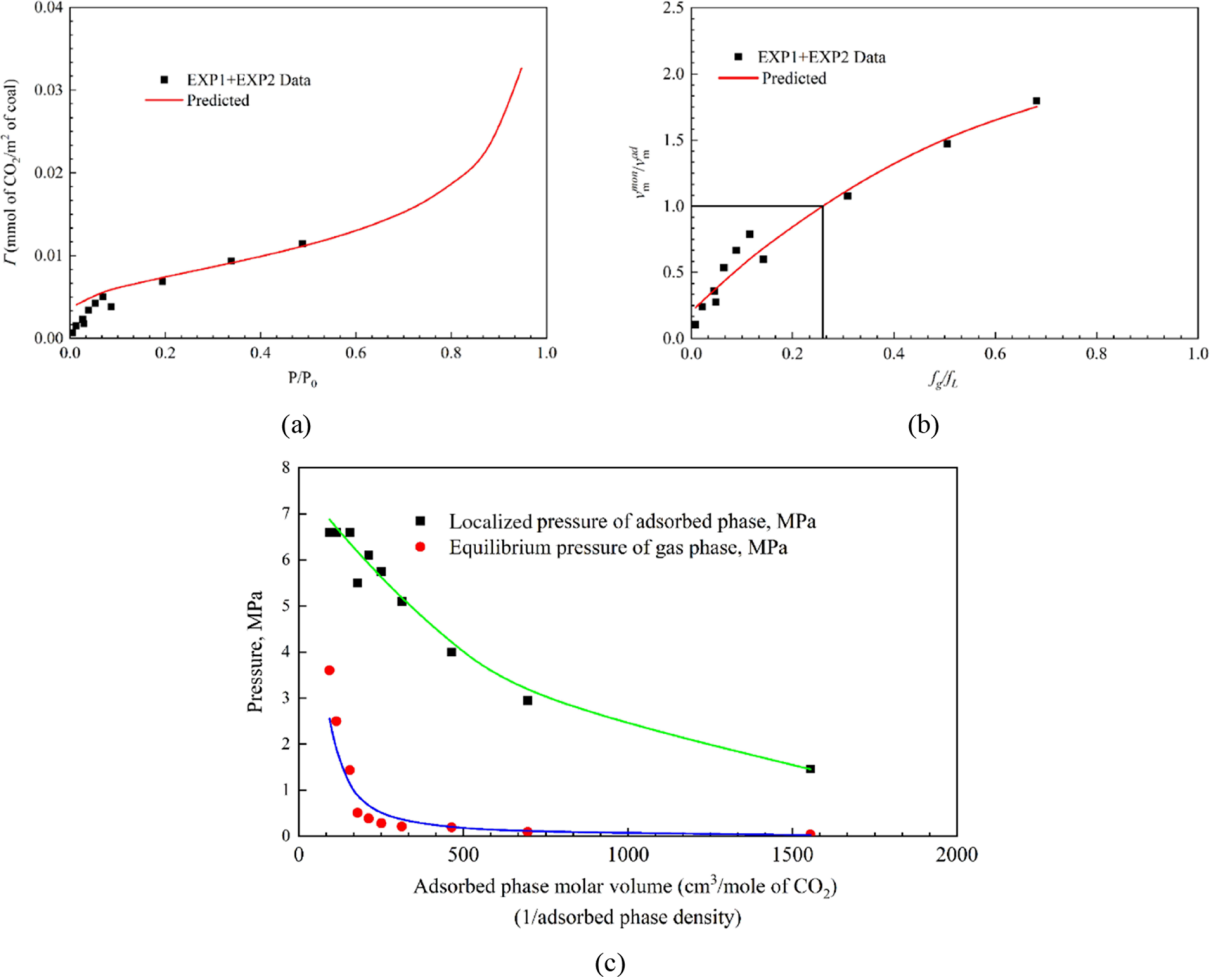
(a) Characteristic curve I, based on potential theory and surface force of attraction. Calculated values of CO_2_ adsorption using eq 13 are plotted against the experimental results obtained from the EMB1 coal specimen. (b) Characteristic curve II, based on molar volume and fugacity of adsorbed and free phase of CO_2_ (EMB coal core specimen with 50 mm dia. and 60 mm length). (c) Calculated localized pressure at the interface of adsorbed phase and measured equilibrium pressure plotted against varying molar volume or adsorbed phase density (Solid lines are trendlines).

The curve is constructed based on a microscopic point of view, e.g., one molecule of CO_2_ attracted by a large body of coal (the potential theory of Polanyi, eq 13). The parameter ‘*C*’ in eq 13 is related to the van der Waals force between coal and CO_2_ molecule or the Hamaker constant. It is in the order of 10^–26^ Jm^3^/mol assuming only van der Waals forces acting between a small molecule and a large surface.^49,53^ The experimental and predicted results of Γ vs  are presented in Figure 6a. The best fit was obtained for *C* = 1 × 10^–27^ Jm^3^/mol. Therefore, it supports the hypothesis that van der Waals/London dispersion forces (surface potential) act between CO_2_ molecules and the bituminous coal surface at such low, subcritical pressure injection.

#### Characteristic Curve II

The characteristic curve assumes a combination of molecular spread (Langmuir-type liquid-like monomolecular layer) and clustering (BET-type liquid-like multiple layers) on a surface. The current study formulated an empirical relationship to view CO_2_ adsorption as a function of change of state. Figure 6b presents the plot of molar volumes of adsorbed phases against fugacity ratio of adsorbed phase and free phase of CO_2_. The *y*-axis shows the ratio of adsorbed phase molar volume and molar volume required to complete the monolayer . The molar volume calculations have been discussed in the Supplementary section. The multilayer builds up occurs when . Below this value, the adsorbed phase molar volume is close to the gas phase molar volume, and above this value, the molar volume is equal to the liquid molar volume of CO_2_ at the adsorbed phase. The *y*-axis values can also be seen as the ratio of the adsorbed phase density of CO_2_ at given equilibrium pressure and density of CO_2_ at complete monolayer coverage. The advantage of this expression is that it depicts the changing adsorbed phase density upon increasing equilibrium pressure and monolayer coverage in a single plot. When the fugacity of the adsorbed phase is equal to the fugacity of the gas phase of CO_2_ in the manometric cell, the sorption process attains equilibrium. The calculated molar volume (*v*_m_^ad^) was used to predict the localized pressure created at the interface of the adsorbed phase and the surface of the coal by employing Peng–Robinson equation of state. Figure 6c shows the gradual increase in the localized pressure upon increasing gas phase pressure. The liquid-like density forms when the localized pressure approaches to 6.1 MPa. This indicates that adsorption inside the matrix pores increases CO_2_ density and drive diffusion in the coal matrix, which is evident in the hysteresis gap, observed in the adsorption–desorption isotherm patterns (Figure 2a,b,d). The pressure dependent gas transport in slit-nanopores is relevant to bituminous coals where the narrow pore widths increase the concentration flux and increases the proportion of the adsorbed molecules.^68^

## Conclusions

The focus of this study was to improve fundamental understanding of low-pressure injection and adsorption–desorption behavior of CO_2_ in shallow-depth coal seams. Different sizes of intact bituminous coal cores and powdered coal samples were tested in laboratory to estimate their CO_2_ sorption capacities. The following conclusions are reached:

(i) Maximum theoretical adsorption capacity of the intact bituminous samples, obtained from the 30 m deep seam, up to 0.5 MPa injection pressure, was estimated between 35.6 and 37.9 g of CO_2_/kg of coal. However, at injection pressure up to 4 MPa, the maximum capacity was 55.4 g of CO_2_/kg of coal.
(ii) Sample mass, after the CO_2_ injection tests, was higher in larger samples than that of the smaller samples. This highlights the importance of micropore network volume on CO_2_ adsorption in intact bituminous coals, which is available at higher quantity in larger coal samples. The time required to reach equilibrium increased as sample size increased. The study also revealed that a longer period of coal-CO_2_ interaction resulted in a higher amount of CO_2_ adsorption.
(iii) The energy of adsorption values (−15 to −20 kJ/mol) observed in this study suggested physical adsorption of CO_2_ at low, subcritical pressures. Adsorption was spontaneous that increased with injection pressure and reversed with reduction of pressure. However, release of CO_2_ experienced a time lag during desorption tests. Adsorption–desorption hysteresis gaps widened with the release of gas pressure suggesting that the trapped/stored CO_2_ in the cores was not readily released. The observed gap, under similar thermodynamic equilibrium conditions, was wider in larger samples than the smaller ones indicating higher amount of residual CO_2_ storage in the larger cores at lower, subcritical pressure conditions. The residual amount of entrapped CO_2_ in a specific coal sample was higher, when it was submitted to a higher pressure suggesting that pore entrapment increases with increasing pressure.
(iv) The preferential sorption experiment showed that intact bituminous coals, obtained from the shallow-depth seam, possess greater affinity to CO_2_ than CH_4_ even at a low injection pressure, e.g., ≤0.5 MPa.
(v) CO_2_ adsorption on the intact bituminous coal samples exhibited the type II isotherm pattern (BET isotherm that also including the Langmuir isotherm) of the IUPAC classification, whereas the adsorption–desorption hysteresis pattern was of Type H3 (representing pore-diffusion or condensation). Evaluation of Langmuir and BET models confirmed the above description since the adsorption data fitted better to BET than the Langmuir isotherm model.
(vi) The analysis of coal–CO_2_ interaction via characteristic curves suggested the presence of van der Waals/London dispersion forces (potential theory of adsorption) between coal surfaces and CO_2_ gas molecules. Empirical relationships based on changing state functions of the adsorbed phase ascertained the concept of CO_2_ adsorption inside matrix pores leading to increased CO_2_ density and pore diffusion. This was also evident through the hysteresis gap and the residual amount of entrapped CO_2_ in the intact core samples.

The findings of this study support the utility of shallow-depth coal seams for CO_2_ storage.

## Supporting Information Available

He-pycnometer method to determine void volume of the reference cell and the empty cell, void volume approximation of the sample cell including a coal sample, modified He-pycnometry volume calculation method, CO_2_ adsorption measurements, calculation procedures for measuring the amount of adsorbed CO_2_ in coal samples, CO_2_ desorption experimental procedures, calculation methods for the adsorption characteristics curve, and calculated values for the characteristic curve II (PDF)

## Notes

The data presented in this article are available via request to the corresponding author.
