# Supplementary material for: Low Subcritical CO2 Adsorption–Desorption Behavior of Intact Bituminous Coal Cores Extracted from a Shallow Coal Seam
Source: Langmuir. 2023 Jan 20;39(4):1548–61. doi: 10.1021/acs.langmuir.2c02971 (PMC9893810; doi:10.1021/acs.langmuir.2c02971)
Supplement: Supplementary file 1 — la2c02971_si_001.pdf [file la2c02971_si_001.pdf]

Supporting Information

Low subcritical CO_2 adsorption-desorption behaviour of intact bituminous coal cores extracted from a shallow coal seam

Shakil A. Masum*, Sivachidambaram Sadasivam, Min Chen, Hywel R. Thomas

Geoenvironmental Research Centre (GRC), School of Engineering, Cardiff University, The Queen's Buildings, The Parade, Cardiff, CF24 3AA, United Kingdom

** Corresponding author: masumsa1@cardiff.ac.uk*

Table of Contents

He-pycnometer method to determine void volume of the reference cell and the empty cell S2

Void volume approximation of the sample cell including a coal sample S3

Modified He-pycnometry volume calculation method S4

CO_2 adsorption measurements S4

Calculation procedures for measuring the amount of adsorbed CO2 in coal samples S6

CO_2 desorption experimental procedures, S7

Calculation methods for the adsorption characteristics curve S7

References S10

He-pycnometer method; determining the void volume of the reference cell (V_{rc}) and the empty sample cell (V_{sc})

The void volumes of the reference and sample cells were determined using the gas expansion method and the Boyle's variant of the gas law (PV=ZnRT). The procedures are outlined below.

(i) A vacuum pump was used to evacuate the entire adsorption cell apparatus as well as the gas pipelines for 24 hours. Before the test, the tubing that connects the system to the helium gas cylinder was vacuumed for 15 minutes.

(ii) The calibration cell (known volume, the volume of calibration cell V_{cc}= 0.0004892 m^3) was connected to the gas line, and the calibration cell temperature was maintained at the same temperature as the water tank (298.15 K). To ensure individual isolation, the valves V1, V2, and V3 (Figure 1) that connected the calibration cell (CC), reference cell (RC), and sample cell (SC) were initially closed.

(iii) The calibration cell (CC) was injected with a known volume of He gas (equal to the V_{cc}) and the pressure data were recorded. Although the pressure readings were stabilised after 120 seconds, around 20 minutes of readings were allowed to monitor any pressure variations caused by indeterminate errors (e.g., leaks, temperature fluctuations). The pressure values were abbreviated as,P_{cc}.

(iv) After recording the P_{cc}, valve V1 was opened to expand the He gas into the reference cell (RC) (Figure 1). The pressure readings (referred to as $P_{cc} + P_{rc}$) were recorded to determine the volume of the reference cell (V_{rc}).

(v) The V2 was then opened to allow the He gas to be expanded into the sample cell (SC). The pressure values (referred to as $P_{cc} + P_{rc} + P_{sc}$) recorded after 20 minutes were used to calculate the sample cell volume (V_{sc}).

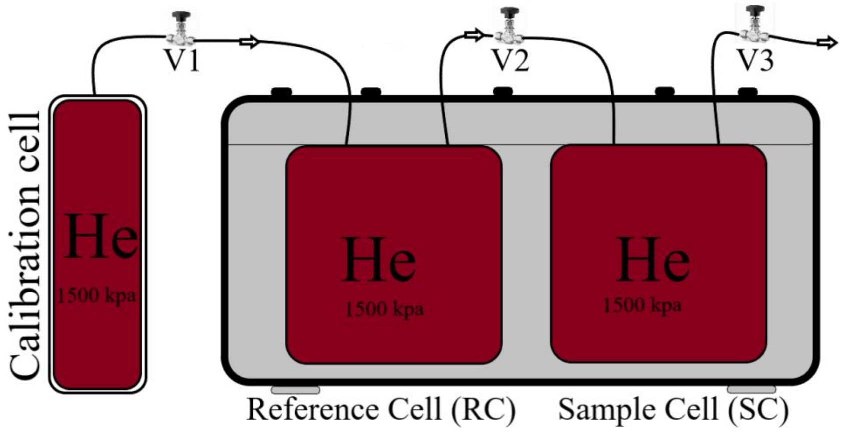

Figure 1. Schematic of He-volume measurement method. Both the reference cell (RC) and the sample cell (SC) are empty

(vi) When the preceding test was completed. To prepare the system for the next injecti on cycle, the calibration cell (CC) and the helium gas line from the cylinder, RC, a nd SC were evacuated through V3 with a vacuum pump for about 10 minutes.

To eliminate any uncertain experimental mistakes, the aforementioned method from I to (vi) was repe ated three times.

Void volume approximation of the sample cell including a coal sample (V_{scv})

Section 2.2 of the main text describes the sample loading and preparation procedures. The experimental approach for calculating the void volume of the sample cell is the same as t he procedures outlined in the section above, from (i) to (v). The only difference was that this time the sample cell (SC) was loaded with a core sample, as seen in Figure 2. The sample cell volume (V_{scv}) was calculated using the stable pressure value measured as $P_{cc} + P_{rc} + P_{scv}$. The volume calculation method is detailed in the following section.

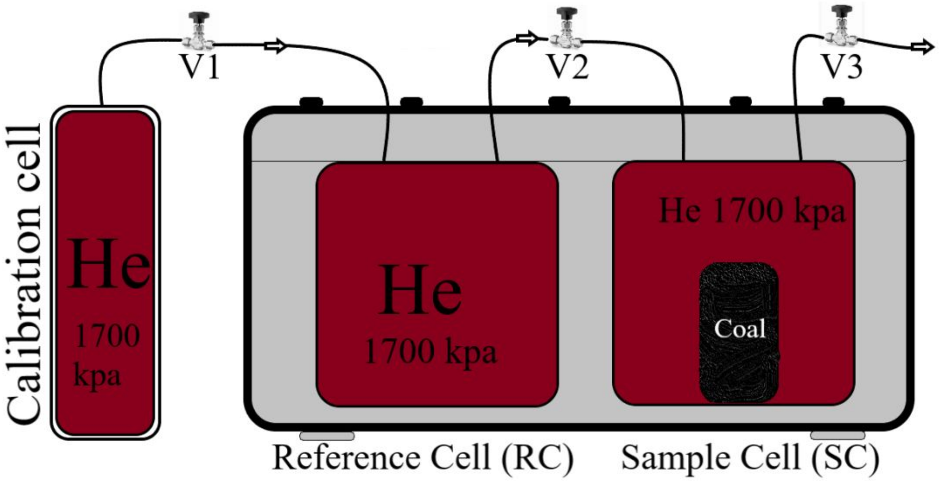

Figure 2. Schematic of He-volume measurement method. The reference cell (RC) is empty, but the sample cell (SC) is loaded with a coal sample.

Modified He-Pycnometry volume calculation method

Using the data generated (pressure values) from the He-Pycnometry methods mentioned in the preceding section, the gas law for real/non-ideal gases, Equation (1), was used to compute the volume of the reference cell (V_{rc}) and sample cell (Vsc). The P-V-T behaviour was considered to reflect the effects of real gas on the molar volume. The following is the calculating technique.

Step 1: To calculate the number of moles injected into the calibration cell.

$$n_{He} = \frac{P_{cc}V_{cc}}{ZRT} \quad (1)$$

where, n_{He} = The number of moles of He, mol, P_{cc} = Pressure measured from CC, Pa, V_{cc} = Volume of CC =0.0004832 m^3, $Z =$ Compressibility factor of He, and R = Universal gas constant = 8.314 Pa m^3/mol/°K

For real gases, the ideal gas law is modified using the compressibility factor to provide a more accurate forecast of cell volume (Smith et al., 2018, Rao, 1997). For the compressibility factor (Z) values, the cubic version of the Peng-Robinson equation of state (PR-EoS) was solved. The values were then compared to compressibility factor (Z) charts to eliminate the deviation error caused by polar gases such as He (Elliot and Lira, 2012; Smith et. al., 2018; Rao, 1997). To calculate the amount of moles, the Z values were inserted in Equation (1).

Step 2: The volume, V_{rc}, was calculated by substituting the number of moles obtained from the foregoing calculations (n_{He}) and the pressure values of $(P_{cc} + P_{rc})$ from section 1.1 in Equation (2). The compressibility factor was estimated for the corresponding measured pressure values of $(P_{cc} + P_{rc})$. The reference cell volume was obtained by subtracting V_{cc} from V_{cc}.

$$V_{cc} + V_{rc} = \frac{n_{He}ZRT}{p_{cc} + p_{rc}} \tag{2}$$

where, V_{cc} = volume of calibration cell, m^3, V_{rc} = volume of the reference cell, m^3, and P_{cc}+P_{rc} = pressure measured when the He was in the calibration cell+ reference cell, Pa.

Step 3: Similar procedures, described in Step 2, were followed to calculate the empty sample cell volume (V_{sc}) (Equations 3 and 4). As the $(V_{cc} + V_{rc})$ was known from steps 1 and 2 and it can be subtracted from $(V_{cc} + V_{rc} + V_{sc})$ to determine the empty sample cell volume (V_{sc}). The sample cell volume with a sample loaded was determined using the same steps as 1 to 3 and referred to as V_{scv}. $(V_{rc} + V_{scv})$ is alluded to as v_d (void volume) in the main text (Equations (1) and (2) of the main text).

$$V_{cc} + V_{rc} + V_{sc} = \frac{n_{He}ZRT}{p_{cc} + p_{rc} + p_{sc}} \tag{3}$$

$$V_{cc} + V_{rc} + V_{svc} = \frac{n_{He}ZRT}{p_{cc} + p_{rc} + p_{scv}} \tag{4}$$

$$(V_{rc} + V_{scv}) = v_d = \frac{n_{He}RTZ_{He}}{P_{He}} \tag{5}$$

where, V_{cc} = Volume of calibration cell, m^3, V_{rc} = Volume of reference cell, m^3, V_{sc} = Volume of sample cell, m^3, V_{scv} = Volume of sample cell with sample, m^3, P_{cc}+P_{rc}+P_{sc} = Pressure measured when the He was in the calibration cell + reference cell + empty sample cell (Figure 2), Pa, and P_{cc}+P_{rc}+P_{scv} = Pressure measured when the He was in the calibration cell + reference cell + sample cell with sample loaded (Figure 2), Pa.

CO_2 adsorption measurements (Section 2.3)

CO_2 gas adsorption studies were carried out in the same manner as helium gas expansion experiments. By measuring the increasing or decreasing adsorptive gas pressure, the amount of CO_2 adsorbed or desorbed was estimated. The steps are outlined below.

(i) In the sample cell, a known mass of coal sample (SC) was placed. Before the test, the entire system was vacuumed for 24 hours, and the pipeline connecting the system to the CO_2 gas cylinder was evacuated for 15 minutes.
(ii) The valves V1, V2, and V3 (Figure 3) were closed to isolate the reference and sample cells. A CO_2 vaporiser was employed to inject the CO_2 in the vapour phase from the cylinder.
(iii) By opening the valve V1, a known quantity (n moles) of CO_2 was fed into RC (Figure 3). The volume of the RC can be calculated using the He-pycnometry volume measurements described in the preceding section.

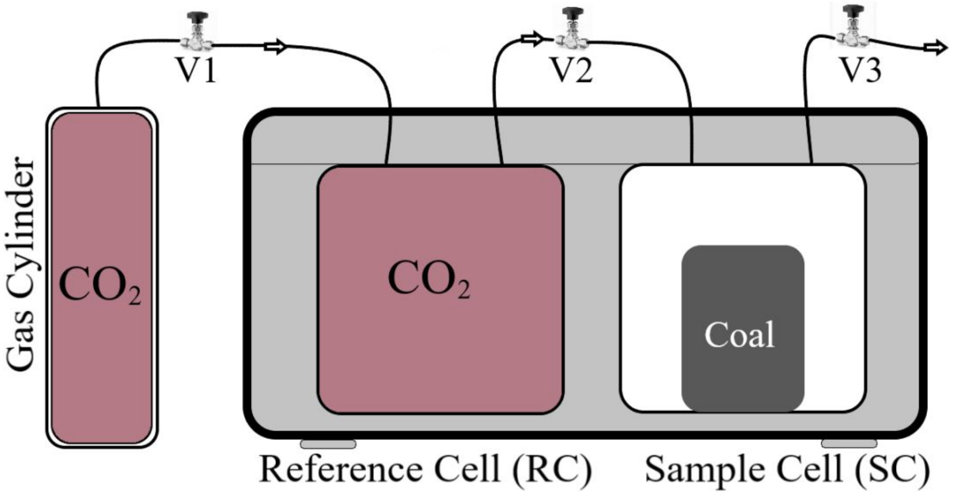

Figure 3. CO_2 adsorption measurement. The reference cell (RC) is filled with known amount of CO_2 while the sample cell (SC) is free of CO2.

(iv) The gas pressure of RC was measured after filling it with a specified volume of CO_2. The injected gas's molar volume was determined using the gas law. The following section explains the calculation strategy. The gas was then expanded to the SC by opening the valve V2 (Figure 4).

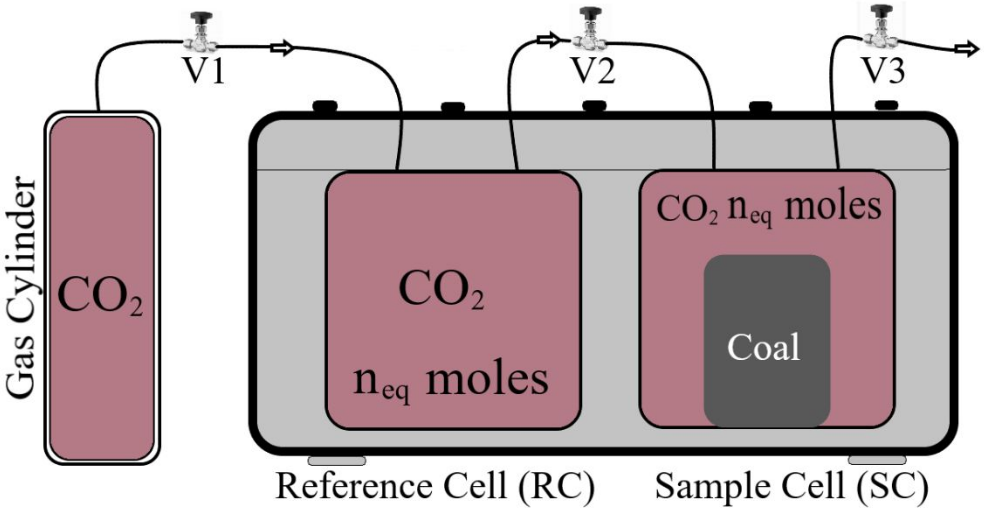

Figure 4. CO_2 adsorption measurement. The reference cell (RC) is filled with CO2 and a known amount of CO_2 gas is expanded into sample cell (SC).

(v) After expanding the gas into SC, the gas pressures in the RC and SC were continually monitored and recorded by an application software provided by the GDS Instruments, UK. The adsorbate gas pressure is decreased in proportion to the rate of adsorption. The sorptive gas was allowed to equilibrate with the sorbent, and the equilibrium gas pressure was identified when the gas pressure values were constant for a minimum of 4 hours. The amount of CO_2 adsorbed was determined by calculating the difference between the number of moles in the gas phase at the beginning of the stage (n moles) and the number of moles at the equilibrium stage (n_{eq} moles).

(vi) The above steps were repeated in order to inject CO2 in phases at injection pressures ranging from 0.1 MPa kPa to 4.0 MPa.

(vii) The preferential adsorption tests were carried out with an 80% CO2 and 20% methane gas mixture, and the experimental protocols were the same as those described before. To confirm the preferential sorption behaviour of the coal specimens, an Emmerson X-stream gas analyser was coupled in the gas line and located before the reference cell to quantify the change in volume percent.

Calculation procedures for measuring the amount of adsorbed CO_2 in coal samples

The number of moles adsorbed in a kg of coal was obtained by calculating the difference between the number of moles in the gas phase at the beginning of each stage and the number of moles in the gas phase at the time of the equilibrium (Equation 5).

$$\frac{n_{CO_2ad.}}{kg\ of\ adsorbent} = \frac{(n_{CO_2rc_0} + n_{CO_2sc_0}) - (n_{CO_2[rc+sc]_{equ}})}{kg\ of\ adsorbent} \quad (6)$$

where, $\frac{n_{CO_2ad.}}{kg\ of\ adsorbent}$ = Moles adsorbed CO_2 per kg mass of adsorbent, mol of CO_2/kg, $n_{CO_2rc_0} + n_{CO_2sc_0}$ = Number of moles in RC+SC at the beginning of each stage, and $n_{CO_2[rc+sc]_{equ}}$ = Number of moles in RC+SC at the end of each stage (equilibrium).

The number of moles of CO_2 in RC and SC was calculated using the gas law.

$$n_{CO_2rc_0} = \frac{P_{rc}V_{rc}}{Z_{rc}RT}\ ;\ n_{CO_2sc_0} = \frac{P_{sc}V_{sc}}{Z_{sc}RT};\ n_{CO_2[rc+sc]_{equ}} = \frac{P_{rc+sc}V_{rc+sc}}{Z_{rc+sc}RT}$$

where, $n_{CO_2rc_0}$ = Number of moles of CO_2 in RC at the beginning of each stage, mol, $n_{CO_2sc_0}$ = Number of moles of CO_2 SC at the beginning of each stage, mol, $n_{CO_2[rc+sc]_{equ}}$ = Number of moles at the end of each stage (equilibrium). mol, P_{cc}+P_{rc}+P_{sc} = Pressure in RC, SC and [RC+SC], Pa, V_{rc}, V_{sc} and $V_{(rc+sc)}$ = Volume of RC, SC and [RC+SC] with coal sample loaded respectively, m^3, Z_{rc}, Z_{sc} and $Z_{(rc+sc)}$ = Compressibility factor for RC, SC and [RC+SC], respectively.

The P-V-T behaviour was predicted using Peng-Robinson equation of state (Z values) (Equation 6). The Peng-Robinson is a modified version of the Redlich-Kwong equation of state (Rao, 1997; and Elliott and Lira, 2012).

$$P = \frac{RT}{V-b} - \frac{a(T)}{V(V+b)+(V-b)} \quad (7)$$

In which, $a(T) = \alpha a(T_c)$, $a(T_c) = 0.45723553\frac{\alpha R^2T_c^2}{P_c}$, $\alpha = [(1+\varkappa(1-\sqrt{\frac{T}{T_c}})\ ]^2$,$\varkappa = 0.37464 + 1.54226\ \omega - 0.26992\omega^2$, $b = 0.07779607\frac{RT_c}{P_c}$, and

$$Z^3 - (1-B)\ Z^2 + (A-3B^2-2B)\ Z - (AB - B^2 - B^3) = 0. \quad (8)$$

In which, $Z = \frac{PV}{RT}$, $A \equiv \frac{aP}{R^2T^2}, B = \frac{bP}{RT}, b\rho = \frac{B}{Z}; \frac{a\rho}{RT} = \frac{A}{Z}$

where, Z = Compressibility factor, P = Equilibrium Pressure, V = Cell Volume, R = Universal Gas constant, T= Temperature, P_c = Critical pressure of CO_2, T_c = Critical temperature of CO_2, ρ = Density $a, \alpha, \varkappa$ = PR-EoS parameters, b = Dimensional PR-EoS parameter, ω = Acentric factor for CO_2.

The non-dimensional form of the PR-EoS (Equation 8) was solved to determine the molar volume of the pressures recorded in reference cell. The Z must be solved for CO_2 vapour/liquid roots to determine the molar volumes of the corresponding states.

CO_2 Desorption experimental procedures

CO_2 desorption experiments were conducted in the same manner as stated in Section 2.2. After the final stage of the adsorption experiment is complete, the valve V2 is closed to isolate the SC from the RC and the pressure in the RC is decreased. Then, valve V2 was opened to connect the RC and SC and enable for equilibrium to be achieved. Pressure increased gradually during desorption when CO_2 molecules were released from the coal surfaces. After achieving equilibrium, the procedures were repeated.

Every ten seconds, the change in gas phase pressure during adsorption and desorption was recorded and used to determine the adsorption and desorption rates.

Calculation methods for the adsorption characteristic curve (Section 4.4)

Characteristic curve II:

The molar volume of the adsorbed phase of CO_2 was calculated using Equation (9).

$$v_m^{ad}(\frac{m^3}{mol}) = \frac{V^a\ (volume\ available\ for \frac{CO_2}{kg}\ of\ coal)}{n\ (\frac{mol\ adsorbed}{kg\ of\ coal})} \quad (9)$$

where, V^a = Volume available for CO_2 in per kg of coal = Void volume of the sample + maximum volume can occupy on the external surface area (assuming, no multilayer on the surface) + connected and unconnected pore volume (1.5% of the bulk volume of the sample), and v_m^{ad} = Molar volume of adsorbed phase, m^3/mol.

Example calculation:

V^a = 6.8966×10^{-05} m^3/kg is calculated by summing up (i) measured void volume of the sample by He-pycnometer method (9.17814×10^{-06} m^3), (ii) maximum volume occupied on the external surface area (calculated using effective contact area of 0.52nm^2 (Atkins et al., 2017) of CO_2 and effective area occupied by CO_2 molecule on coal 0.260 nm^2 (calculated after Pennell, 2002)- this value is negligible, and (iii) the microporous void volume measured using X-ray CT (X-ray computed tomography), which is 1.5% of the bulk volume of the coal. The bulk volume of coal is 0.000111 m^3/0.1404 kg of coal. The calculated total volume was propagated to a kg of coal as shown in Equation (9a) and substituted in Equation (9).

$$V^a = \frac{9.17814 \times 10^{-06}\, m^3 + (0.015 \times 0.000111)\, m^3}{0.1404\, kg} \quad (9a)$$

To achieve the molar volume or adsorbed phase density, the amount adsorbed for a given equilibrium pressure was replaced into equation (9). Then, $\frac{v_m^{mon}}{v_m^{ad}}$ ratio is calculated using the molar volume of monolayer adsorption (v_m^{mon} = 0.000167 m^3/mol) calculated from BET (n_{mon}) isotherm model.

The fugacity values gas phase (f_g) and adsorbed phase (f_g) were calculated using Peng-Robinson equation of state for the corresponding gas pressure.

For example, consider the first data point on the *characteristic curve II*, the adsorbed phase molar volume of $v_m^{ad} =$ 0.00155 m^3/mol CO_2 would yield an adsorbed phase fugacity of f_a =1.35 MPa. The corresponding equilibrium gas phase fugacity is f_g= 0.035 MPa. The ratio of $\frac{f_g}{f_a}$ is 0.026. The ratio of $\frac{v_m^{mon}}{v_m^{ad}}$ is 0.11. Thus, the first data point constructed for the *characteristic curve II.*

The adsorbed phase fugacity is limited to the liquid phase fugacity of 4.29 MPa. When fugacity ratio of gas phase is equal to 4.29 the adsorption reaches infinite, and condensation sets in. The calculated values for the *characteristic curve II* are presented in Table S1.

Table S1. Calculated values for the *characteristic curve II*.

f_g/f_a (x-axis)	*fa* (Fugacity of adsorbed phase CO_2), MPa	f_L (Fugacity of liquid phase CO2), MPa	f_g (fugacity of gas phase), MPa	Equilibrium Pressure, MPa	amount of CO2 adsorbed, g/kg of coal	n, Amount of CO2 adsorbed, mol/kg	Va/kg, Void volume, m^3	V^m_{ad}, molar volume of adsorbed Phase, m^3/mol	V_L, molar volume of liquid CO_2, m^3/mol	V^{mom}, molar volume required to complete monolayer, m3/mol	V^{mon}/V^m_{ad}
0.026	1.35	4.29	0.035	0.034714	1.95	0.044362	6.9E-05	0.0016	0.00007	0.000167	0.11
0.037	2.5	4.29	0.091	0.09152	4.37	0.09918	6.9E-05	0.00070	0.00007	0.000167	0.241
0.061	3.17	4.29	0.19	0.193561	6.54	0.148654	6.9E-05	0.00046	0.00007	0.000167	0.361
0.05	3.76	4.29	0.21	0.210213	5.00	0.113643	6.9E-05	0.00061	0.00007	0.000167	0.28
0.068	4.04	4.29	0.28	0.280874	9.71	0.22066	6.9E-05	0.00031	0.00007	0.000167	0.54
0.091	4.17	4.29	0.38	0.386071	12.13	0.275542	6.9E-05	0.00025	0.00007	0.000167	0.67
0.125	3.94	4.29	0.498	0.508099	14.33	0.325506	6.9E-05	0.00021	0.00007	0.000167	0.79
0.307	4.31	4.29	1.328	1.433396	19.6	0.44534	6.9E-05	0.000155	0.00007	0.000167	1.08
0.503	4.31	4.29	2.17	2.49733	26.72	0.607014	6.9E-05	0.000114	0.00007	0.000167	1.5
0.68	4.31	4.29	2.92	3.603275	32.64	0.741704	6.9E-05	0.0000929	0.00007	0.000167	1.8

References

Atkins P, De Paula J, Keeler J. (2017). *Atkins' physical chemistry* (11th ed.). Oxford University Press.

Elliott JR, Lira CT. (2012). Introductory Chemical Engineering Thermodynamics. Upper Saddle River, NJ: Prentice Hall PTR.

Pennell KD. (2002). 2.5 Specific Surface Area. In Methods of Soil Analysis (eds J.H. Dane and G. Clarke Topp). https://doi.org/10.2136/sssabookser5.4.c13

Rao YVC. (1997). *Chemical Engineering Thermodynamics*. Universities Press (India). ISBN 81-7371-048-1.

Smith JM, van Ness HC, Abbott MM, Swihart MT. (2018) Introduction to Chemical Engineering Thermodynamics, 8th edition, McGraw-Hill, New York.
